# Techniques of analysing residual alveolar ridge width before dental implant placement: A comparative clinico-imaging study

**DOI:** 10.6026/973206300213921

**Published:** 2025-10-31

**Authors:** Malav Sunilbhai Sheth, Nikunj Harikrushn Prajapati, Abhishek Singh Payak, Priti P Shah, Devanshi Vaghela, Akhila S Aboobacker

**Affiliations:** 1 Department of Periodontics and Implantology, Siddhpur Dental College and Hospital, Patan, Gujarat, India; 2 Department of Orthodontics and Dentofacial Orthopedics, Siddhpur Dental College and Hospital, Patan, Gujarat, India; 3 Department of Dentistry, Goverment Medical College, Ratlam, Madhya Pradesh, India; 4 Department of Oral Medicine and Radiology, Faculty of Dental Science, Dharamsinh Desai University, Nadiad, Gujarat, India; 5 Department of Oral and Maxillofacial Surgery, Karnavati School of Dentistry, Karnavati University, Gandhinagar, Gujarat, India; 6 Department of Prosthodontics, KMCT Dental College, Calicut, Kerala, India

**Keywords:** Dental implants, alveolar ridge width, cone-beam computed tomography, panoramic radiography, pre-surgical planning, measurement accuracy

## Abstract

Accurate assessment of alveolar ridge width is critical for implant planning. Therefore, it is of interest to compare measurements from orthopantomography (OPG) and cone-beam computed tomography (CBCT) with direct intraoperative caliper evaluation in 50 posterior mandibular sites. CBCT values (6.15 ± 1.14 mm) closely matched direct measurements (6.18 ± 1.12 mm) with no significant difference (p=0.68). OPG significantly overestimated ridge width (7.64 ± 1.55 mm; p<0.001). CBCT demonstrated high reliability within ±0.5 mm accuracy in 94% of cases, supporting its role as the gold standard for pre-surgical width analysis in implant dentistry.

## Background:

The replacement of missing teeth with endosseous dental implants has become a cornerstone of modern restorative dentistry, offering high success rates and improved quality of life for patients [1]. The fundamental prerequisite for long-term implant success is achieving primary stability and subsequent osseointegration, both of which are critically dependent on the volume and quality of the available host bone [2]. Following tooth extraction, the alveolar ridge undergoes a physiological process of resorption, which is most pronounced in the first year and leads to a significant reduction in both width (bucco-lingual dimension) and height [3]. Insufficient alveolar ridge width is a common clinical challenge that can compromise ideal implant placement. Accurate pre-surgical assessment of the residual alveolar ridge is therefore not an elective step but an essential component of treatment planning [4]. An adequate bone volume surrounding the implant is necessary to ensure its complete encasement, prevent aesthetic and biological complications such as fenestration or dehiscence, and provide a stable foundation for the final prosthesis [5]. Misjudging the ridge width can lead to the selection of an inappropriately sized implant, increasing the risk of thread exposure, marginal bone loss, and potential damage to adjacent anatomical structures [6]. Historically, clinicians relied on a combination of clinical examination and two-dimensional (2D) radiographic imaging, such as periapical and panoramic radiographs (OPG), for pre-surgical evaluation [7]. While OPGs are valuable for providing a broad overview of the jaws, identifying pathologies, and assessing vertical bone height, they are inherently limited by geometric distortion, magnification, and the superimposition of anatomical structures. Critically, they offer no information on the bucco-lingual dimension of the alveolar ridge, which is a three-dimensional (3D) structure [8].

The advent of cone-beam computed tomography (CBCT) has revolutionized dental implant planning by providing high-resolution, multi-planar 3D images of the maxillofacial skeleton at a relatively lower radiation dose compared to conventional medical CT [9, 10]. CBCT allows for precise, cross-sectional visualization of the proposed implant site, enabling accurate linear measurements of bone width and height, assessment of bone density, and clear identification of vital structures like the inferior alveolar nerve and maxillary sinus [11]. Several studies have validated the high accuracy of linear measurements made on CBCT scans [12, 13]. Despite the well-documented advantages of CBCT, the use of OPG as a preliminary or sometimes sole imaging tool persists in some clinical settings due to its lower cost, wider availability, and minimal radiation exposure. This practice creates a potential for diagnostic error. While the superiority of CBCT is generally accepted, there is a continuous need for clinical studies that directly quantify the discrepancy between these modalities and the true anatomical dimensions measured during surgery. Such data reinforces evidence-based clinical guidelines and educates practitioners on the potential pitfalls of relying on inadequate diagnostic information. This study, therefore, fills a crucial gap by performing a direct, *in vivo* comparison of measurements from OPG and CBCT with the surgical gold standard. Therefore, it is of interest to quantitatively compare the accuracy and reliability of residual alveolar ridge width measurements obtained from panoramic radiography and cone-beam computed tomography against direct intraoperative measurements in patients scheduled for dental implant placement. The null hypothesis was that there would be no significant difference in the measurements obtained from the three methods.

## Materials and Methods:

## Study design and sample:

A total of 50 consecutive patients (28 male, 22 female; mean age 48.6 ± 9.2 years) scheduled for single-tooth implant placement in the posterior mandible (premolar and molar regions) between June 2023 and May 2024 were enrolled. Sample size was calculated based on a power analysis (α=0.05, power=0.90) to detect a clinically significant difference of 0.5 mm in ridge width measurements.

## Inclusion and exclusion criteria:

Patients were included if they were over 18 years of age, required a single implant in a healed edentulous site (minimum 6 months post-extraction), and were in good general health. Exclusion criteria included: systemic diseases known to affect bone metabolism (e.g., uncontrolled diabetes, osteoporosis requiring bisphosphonate therapy), active periodontal disease, heavy smoking (>10 cigarettes/day), pregnancy, previous bone grafting at the implant site, or presence of radiographic artifacts that would impede accurate measurement.

## Imaging protocol:

All patients underwent standardized imaging procedures.

[1] Panoramic radiography (OPG): A digital panoramic radiograph was taken for each patient using an Orthopantomograph OP300 machine (KaVo Kerr, Biberach, Germany) with standardized exposure parameters (70 kVp, 10 mA, 14 s). Patients were positioned with the Frankfort horizontal plane parallel to the floor.

[2] Cone-Beam Computed Tomography (CBCT): CBCT scans were acquired using a Planmeca ProMax 3D Mid unit (Planmeca, Helsinki, Finland). The acquisition protocol was standardized with a 9x11 cm field of view (FOV), 90 kVp, 12.5 mA, 0.2 mm voxel size, and an 18-second scan time. Patients were stabilized using a chin rest and head strap to minimize motion artifacts.

## Measurement procedures:

All radiographic measurements were performed by two calibrated examiners, blinded to each other's findings and to the surgical measurements. An average of the two examiners' measurements was used for final analysis.

[1] Radiographic measurements: The CBCT data were imported in DICOM format into PlanmecaRomexis® software (v. 5.1). On the panoramic overview, the intended implant site was identified. A cross-sectional slice perpendicular to the alveolar ridge was selected. The bucco-lingual ridge width was measured 3 mm apical to the most coronal point of the alveolar crest using the software's linear measurement tool. For the OPG, the same software was used to measure the apparent mesio-distal width of the bone at the same vertical level, which is often misinterpreted as a proxy for available bone.

[2] Intraoperative direct measurement (gold standard): During the implant surgery, under local anaesthesia, a full-thickness mucoperiosteal flap was elevated to expose the alveolar ridge. A sterile digital caliper (Mitutoyo, Kawasaki, Japan; accuracy ±0.01 mm) was used to measure the bucco-lingual width of the ridge at the same location as the radiographic measurements (3 mm from the crest). This was performed by the operating surgeon, who was blinded to the pre-surgical radiographic measurements.

## Statistical analysis:

Data were compiled and analysed using IBM SPSS Statistics for Windows, Version 26.0 (IBM Corp., Armonk, NY). Descriptive statistics (mean, standard deviation [SD], minimum, maximum) were calculated for each measurement method. The normality of data distribution was confirmed using the Shapiro-Wilk test. A one-way repeated measures ANOVA followed by post-hoc tests with Bonferroni correction was used to compare the mean ridge widths among the three techniques (Direct, CBCT, OPG). Paired t-tests were also used for direct comparisons between methods. The level of statistical significance was set at p < 0.05. A Bland-Altman analysis was performed to assess the agreement between CBCT/OPG and the direct measurement standard.

## Results:

All 50 patients completed the study protocol. The cohort consisted of 28 males (56%) and 22 females (44%), with an age range of 29 to 67 years. A total of 50 implant sites in the posterior mandible were evaluated. The mean, standard deviation (SD), and range (minimum and maximum) for alveolar ridge width measurements obtained by each of the three techniques are presented in Table 1 (see PDF). The mean width obtained from direct intraoperative measurement was 6.18 ± 1.12 mm, which was almost identical to the mean width from CBCT (6.15 ± 1.14 mm). In stark contrast, the mean width derived from OPG was substantially larger at 7.64 ± 1.55 mm. The standard deviation was also highest for the OPG measurements, indicating greater variability. The results of the comparative statistical analysis are shown in Table 2 (see PDF). The repeated measures ANOVA revealed a highly significant difference among the three measurement techniques (F (2, 98) = 89.4, p < 0.001). Post-hoc comparisons confirmed that there was no statistically significant difference between the direct measurements and the CBCT measurements (mean difference = -0.03 mm, p=0.68). However, the OPG measurements were significantly different from both the direct measurements (mean difference = +1.46 mm, p < 0.001) and the CBCT measurements (mean difference = +1.49 mm, p < 0.001). This indicates a consistent and significant overestimation of ridge dimensions by panoramic radiography. To assess the clinical relevance of these findings, measurements were categorized based on their deviation from the gold standard (direct measurement). A difference of ±0.5 mm was considered a clinically acceptable margin of error for safe implant planning. As detailed in Table 3 (see PDF), CBCT measurements were highly accurate, with 94% of readings falling within this acceptable range. In contrast, only 18% of OPG measurements met this criterion, with the vast majority (82%) deviating by more than 0.5 mm from the actual surgical dimension.

## Discussion:

The primary objective of this study was to evaluate the diagnostic accuracy of OPG and CBCT for determining alveolar ridge width by comparing them to direct intraoperative measurements. The findings of this investigation clearly demonstrate that CBCT is a highly accurate and reliable imaging modality for this purpose, while OPG is prone to significant measurement errors. Our results led to the rejection of the null hypothesis, as a statistically significant difference was found between OPG and the other two methods. The mean ridge width measured on CBCT scans (6.15 mm) was virtually identical to the surgical gold standard (6.18 mm), with a non-significant mean difference of just -0.03 mm. This high degree of accuracy is consistent with a large body of literature. Studies by Takeshita *et al.* [14] and Awasthi *et al.* [15] similarly reported that linear measurements of bone on CBCT scans are dimensionally accurate, with minimal deviation from physical measurements. The inherent technological advantage of CBCT lies in its ability to generate distortion-free, 1:1 scale, multi-planar images, allowing for precise visualization and measurement of the bone in all three dimensions. The clinical implication of this accuracy is profound: clinicians can confidently select an appropriate implant diameter, plan the ideal implant trajectory, and anticipate the need for bone augmentation procedures with a high degree of certainty [11]. In our study, 94% of CBCT measurements were within 0.5 mm of the true width, a margin of error that is highly unlikely to alter the surgical plan. Conversely, our results highlight the severe limitations of panoramic radiography for assessing ridge width. The OPG measurements overestimated the actual ridge width by an average of 1.46 mm, a difference that is both statistically and clinically significant (p<0.001). This overestimation is an artifact of the imaging technique, where the 2D image is a composite of a curved anatomical plane, leading to magnification and distortion that vary across the radiograph [8]. Furthermore, an OPG displays a summative image of all structures in the beam's path, and what is measured is often the wider basal bone rather than the narrower crestal ridge. Relying on such an image for width assessment could lead a clinician to erroneously believe sufficient bone exists, potentially resulting in the selection of an oversized implant. This could precipitate surgical complications such as buccal plate perforation, fenestration, or dehiscence, compromising both the biological and aesthetic outcome of the treatment [5, 6]. Our finding that 82% of OPG measurements were clinically inaccurate (differing by >0.5 mm) strongly supports the recommendation that OPG should not be used for definitive bucco-lingual site assessment in implant planning [7]. The clinical relevance of these findings cannot be overstated. Consider a site with a true ridge width of 5.5 mm. A CBCT would accurately show this, leading the clinician to choose a 3.5 or 4.0 mm diameter implant, ensuring at least 1.5 mm of bone circumferentially. An OPG, by overestimating the width to potentially 7.0 mm, might mislead the clinician into selecting a 5.0 mm implant, leaving dangerously thin buccal and lingual plates and jeopardizing the long-term prognosis [16]. While the superiority of CBCT is clear, its application must be balanced with the ALARA (As Low As Reasonably Achievable) principle for radiation exposure [10]. Guidelines from professional bodies, such as the American Academy of Oral and Maxillofacial Radiology, recommend that CBCT be used when the clinical evaluation and conventional radiography do not provide sufficient information for diagnosis and treatment planning [17, 18]. Our study provides strong evidence that for the specific task of assessing ridge width, conventional radiography (OPG) is indeed insufficient. Therefore, the use of a limited field-of-view (FOV) CBCT, which minimizes the effective radiation dose, is justified and should be considered the standard of care for planning most implant cases. This study has several limitations. It was conducted at a single centre, and the sample was limited to the posterior mandible, which may not be generalizable to the anatomical variations in the anterior maxilla. Furthermore, while the examiners were calibrated, minor inter-operator variability in measurement point selection is always possible. Future multi-centre studies with larger sample sizes and including different areas of the jaws could further strengthen these conclusions and explore the influence of different CBCT machine parameters on measurement accuracy.

## Conclusion:

CBCT offers an accurate and reliable assessment of residual alveolar ridge width, with measurements closely matching direct intraoperative values. In contrast, OPG significantly overestimates ridge dimensions and lacks clinical reliability. Therefore, CBCT should be regarded as the imaging modality of choice for precise implant site evaluation and safe treatment planning.
